# Detection of Early Glaucomatous Damage: Performance of Summary Statistics From Optical Coherence Tomography and Perimetry

**DOI:** 10.1167/tvst.11.3.36

**Published:** 2022-03-30

**Authors:** Emmanouil Tsamis, Sol La Bruna, Ari Leshno, Carlos Gustavo De Moraes, Donald Hood

**Affiliations:** 1 Department of Psychology, Columbia University, New York, NY, USA; 2 Bernard and Shirlee Glaucoma Research Lab, Department of Ophthalmology, Columbia University, New York, NY, USA; 3 Sackler Faculty of Medicine, Tel Aviv University, Tel Aviv, Israel; 4 The Sheba Talpiot Leader Program, Sheba Medical Center Hospital- Tel Hashomer, Ramat Gan, Israel

**Keywords:** glaucoma, glaucoma posterior segment, optical coherence tomography, summary statistics, automated perimetry

## Abstract

**Purpose:**

To evaluate the diagnostic performance of optical coherence tomography (OCT) and visual field (VF) summary statistics (metrics) that are available in OCT and VF reports.

**Methods:**

OCT disc and macular scans and 24-2 and 10-2 VFs were obtained from 56 healthy control (HC) eyes/participants and 61 eyes/patients with 24-2 mean deviation of better than –6 dB. All metrics were obtained from OCT radial, circle, and posterior pole cube scans and 24-2 and 10-2 VFs. Their diagnostic performances were evaluated, in isolation and in combinations. For specificity, the 56 HC eyes were used. For sensitivity, 40 of the 61 patient eyes were deemed likely glaucomatous based on an automated topographic method that evaluates structure–function (S–F) agreement. Any 1 of these 40 eyes not judged as abnormal by any given metric was considered a false negative.

**Results:**

All single OCT and VF metrics misclassified HCs as glaucomatous and missed likely glaucomatous eyes. The best performing single metric was the temporal inferior thickness of the 3.5-mm circle scan, with 96% specificity and 83% sensitivity. Combinations of OCT–OCT and OCT–VF metrics markedly improved specificity. A newly proposed metric that evaluates structure–structure (S–S) agreement at a hemifield level had the highest accuracy. This S–S metric had 98% specificity and 80% sensitivity.

**Conclusions:**

OCT and VF metrics, single or in combinations, have only moderate sensitivity for eyes with early glaucoma.

**Translational Relevance:**

OCT and VF metrics combinations evaluating S–S or S–F agreement can be highly specific, which is an important implication for clinical and research purposes.

## Introduction

The diagnosis of glaucoma has traditionally relied on the assessment of the optic disc and the psychophysical testing of the visual field (VF) to identify characteristic patterns of structural and functional damage. Over the past 30 or so years, standard automated perimetry has become the clinical standard for VF testing.^1^ Likewise, optical coherence tomography (OCT) is increasingly becoming the primary approach to evaluate the optic nerve structure, supplementing optic disc photography and clinical examination.^2^^–^^7^ Commercially available summary statistics from standard automated perimetry and OCT reports are commonly used by clinicians to inform their decision regarding the presence of glaucomatous optic nerve damage.^8^^–^^13^ Such summary statistics are often reported by the manufacturers in a color-coded (traffic light) scheme after a comparison with a normative database. Eventually, a green summary metric is within normal limits, yellow indicates borderline (*P* < 5%), and red is outside normal limits (*P* < 1%). Our focus here is on summary statistics readily available from commercial reports and/or those that can be simply calculated from statistics on these reports. We refer to these available summary statistics as metrics.

Several studies and clinical trials have attempted to define and diagnose glaucoma by using OCT and VF metrics, either in combinations or in isolation.^13^^–^^18^ However, there is little or no consensus as to which is the best approach. For instance, there is evidence that these metrics can miss clear, often local, glaucomatous damage, including damage near fixation.^4^^,^^19^^–^^25^ In addition, a diagnostic evaluation based solely on metrics can fail for other reasons, such as segmentation errors, in the case of OCT, or patient variability with regard to VFs.^20^^,^^26^^–^^31^ In contrast, we and others have argued that it is possible to improve diagnostic performance by taking into consideration the topographical nature of glaucomatous structural damage and/or the relationship between structural and functional abnormalities.^14^^,^^15^^,^^32^^,^^33^ Yang et al.,^14^ for example, reported on the diagnostic performance when the requirement was set for an OCT abnormality to occur in topographically correspondent sectors of the minimum rim width (MRW) and the circumpapillary retinal nerve fiber layer (cpRNFL). Iyer et al.^15^ presented 3 diagnostic criteria, all of which paired quadrant OCT cpRFNL metrics with VF metrics; in particular, the glaucoma hemifield test (GHT) or the pattern standard deviation (PSD), depending on the chosen criterion.

It is difficult to evaluate these competing claims and metrics because the various studies involved used different inclusion criteria and different reference standards for defining glaucoma. The main purpose of the present study was to evaluate the diagnostic performance of proposed OCT and VF metrics, which are readily available in commercial reports and/or easily calculated from these metrics.

## Methods

### Participants

All data included in this study were collected as part of an observational, prospective, case-control study, the Macular Damage in Early Glaucoma and Progression Study (MAPS) (principal investigator, C. Gustavo De Moraes; ClinicalTrials.gov Identifier: NCT02547740). In particular, baseline OCT and 24-2 and 10-2 VF data were obtained from 117 study eyes/individuals: 56 healthy controls (HC) and 61 patients. More than 98% of study visits had OCT scans and both 24-2 and 10-2 VFs acquired on the same date. The remaining had a median difference of 4 days between OCT and VF tests (interquartile range, 2–7 days; range, 1–13 days). All HCs had fundus examination and VFs within normal limits and an intraocular pressure of less than 22 mm Hg. The 61 patients’ eyes were classified as glaucoma or glaucoma suspects based upon the referring glaucoma specialist's interpretation of functional (24-2 and 10-2 VFs) and structural (fundus photos, OCT) information, as well as intraocular pressure and clinical history. Note, however, that the specialist's diagnosis was not used for the purposes of this study. All eyes had a best-corrected visual acuity of better than 20/40, open angles, and 24-2 mean deviation (MD) of better than –6 dB at the first 2 baseline visits. Eyes with high myopia (<−6 diopters) and/or other ocular or systemic conditions that could affect VF or OCT imaging results (e.g., retinal vein occlusion, demyelinating disease) were not part of the MAPS study.

Study procedures followed the tenets of the Declaration of Helsinki and Health Insurance Portability and Accountability Act and were approved by the Institutional Review Board of Columbia University. Written informed consent was obtained from all participants.

### OCT

**Figure 1.**
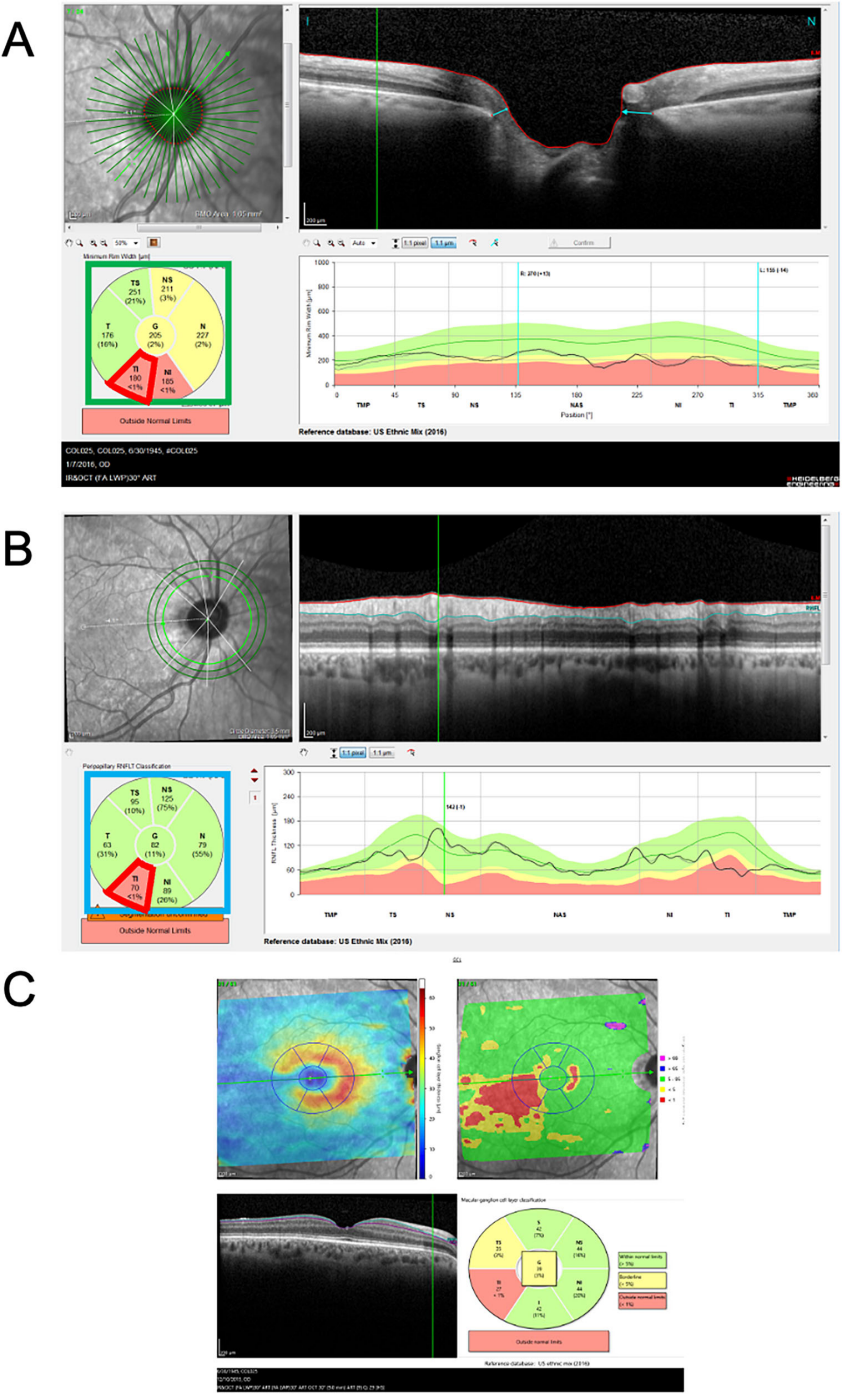
Heidelberg Spectralis’ reports for (A) BMO–MRW, (B) circumpapillary RNFL, and (C) retinal ganglion cell. The level of abnormality from all summary measures shown in the 3 pie charts were exported for the study analysis.

All eyes were scanned with the Spectralis HRA+OCT (Heidelberg Engineering Inc, Heidelberg, Germany) following the Glaucoma Module Premium Edition (GMPE) protocol. As a part of the GMPE protocol, 24 radial scans were acquired over the optic disc, and through these radial scans, the average Bruch's membrane opening (BMO)–MRW was measured for a global (G) and 6 sectoral summary metrics (see Fig. 1A). The manufacturer provides their confidence (or probability) for abnormality with the color scheme described above: that is, green for within normal limits, yellow for borderline (*P* < 5%), and red for abnormal (*P* < 1%). We used these 3 levels, rather than the actual average thickness values. Next, 3 circumpapillary (circle) OCT scans were obtained while centered on the disc with diameters 3.5, 4.1, and 4.7 mm. From each circle scan, the average cpRNFL thickness was measured for the same 7 regions; G and 6 sectors. We used the summary metrics color codes from the small (3.5 mm) (Fig. 1B) and the large circle scans (4.7 mm). The GMPE protocol also provides cube scans of the posterior pole, centered on the fovea and obtained along an axis from the foveal center to the BMO center. Again, G and 6 sectoral metrics were calculated and the usual color codes were extracted for (1) total retinal, (2) RNFL, (3) ganglion cell layer (GCL) (Fig. 1C), and (4) inner plexiform layer (IPL) thicknesses. Note that color codes from total retina and IPL thickness are not available in US commercial devices, without a special (research) license by the manufacturer. Segmentation was not corrected manually so as to be more representative of regular clinical practice. For convenience, a comprehensive list of the metrics and their abbreviations is provided in Supplementary Table S1.

**Figure 2.**
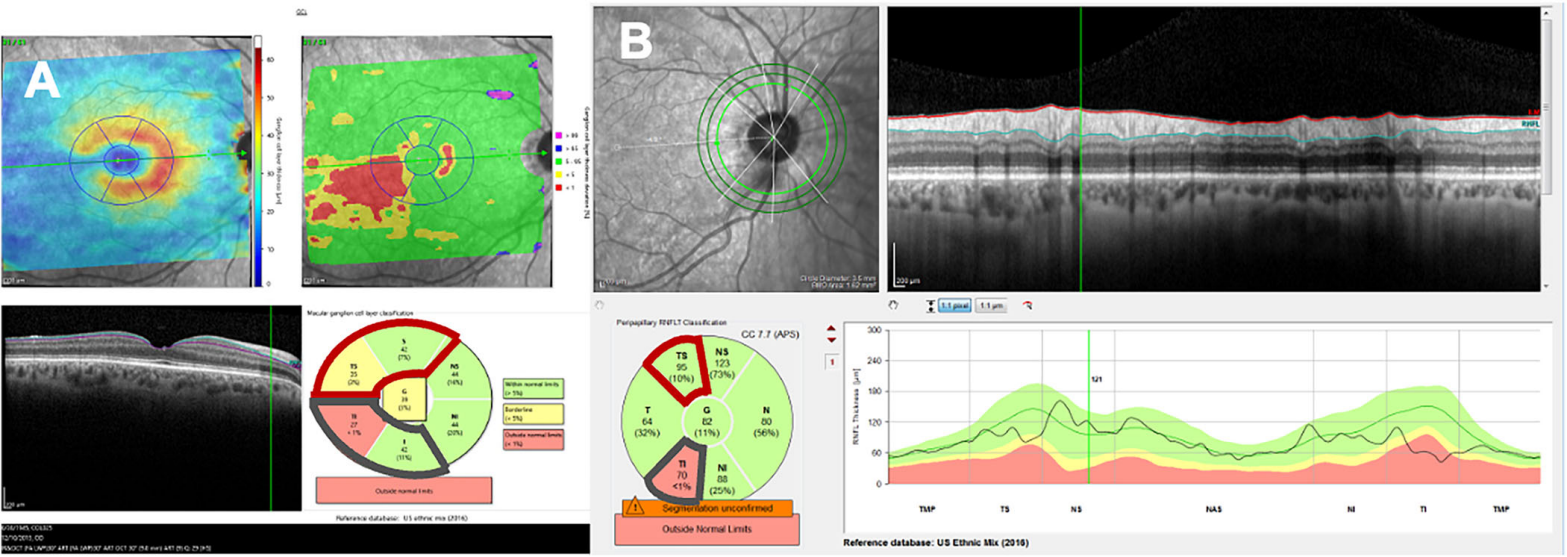
Heidelberg Spectralis’ reports for (A) retinal GCL and (B) cpRNFL. Our proposed S–S metric checks for agreement in abnormality at a hemifield level. An eye is abnormal if either inferior or superior S–S agreement is present. For inferior S–S, the temporal inferior (TI) sector of the cpRNFL (B, in gray) and either the TI or the inferior (I) sector of the GCL (A, in gray) need to be abnormal. For superior S–S, the equivalent sectors from cpRNFL AND at least one from GCL (A and B, in red) need to be abnormal.

We also included a novel structure–structure (S–S) metric, which is based on topographical agreement between GCL and RNFL abnormalities.^20^ For an eye to be abnormal, it had to show both abnormal GCL and RNFL in the superior and/or the inferior retina. In particular, we defined an abnormal inferior S–S agreement as (1) an abnormal temporal–inferior (TI) cpRNFL sector (Fig. 2B, metric highlighted in dark grey) and (2) an abnormal inferior or an abnormal TI GCL sector (Fig. 2A, metrics highlighted in dark grey). Here, we abbreviate this inferior S–S agreement as: [TI_small_ AND (TI_GCL_ OR I_GCL_)]. Similarly, an abnormal superior S–S agreement was defined as: an abnormal temporal–superior cpRNFL sector AND an abnormal temporal–superior or abnormal superior GCL sector: [TS_small_ AND (TS_GCL_ OR S_GCL_)] (Figs. 2A and B, metrics highlighted in dark red). Finally, an abnormal or glaucomatous eye is defined as an eye that has either abnormal inferior S–S agreement OR abnormal superior S–S agreement (the S–S metric).

We evaluated the detection performance of each OCT summary metric in isolation. In addition, we assessed various combinations of OCT metrics (i.e., structural agreement), including our new S–S metric as well as the combination of metrics described by Yang et al.^14^ In particular, we combined small- and large-circle cpRNFL metrics, cpRNFL, and BMO–MRW metrics (including the ones suggested by Yang et al.) and cpRNFL and GCL metrics. For the purpose of this study, each of the metrics was considered abnormal if its value was at a borderline level (*P* < 5%) or less. The results of a similar analysis for a stricter criterion (i.e., abnormal = outside normal limits; *P* < 1%) are provided in Supplementary Tables S2 to S5. In general, the stricter criterion showed worse performance for all metrics.

### Visual Fields

All eyes underwent VF testing with a Humphrey Field Analyzer (HFA, Carl Zeiss Meditec, Inc., Dublin, CA), using the 24-2 and the 10-2 testing patterns (random order of tests, SITA Standard strategy). From the 24-2 single field report the significance (*P* values) of the MD, and the PSD and the categorization of the GHT were obtained, while for the 10-2 report the *P* values of the MD and PSD were used. VF tests were excluded if false positive (FP) responses were greater than 15% or fixation losses were greater than 33%.

Each VF metric alone, as well as all possible combinations (i.e., function–function, F–F, agreement) between them, were evaluated. We also estimated performance measures for the Brusini Glaucoma Staging System (GSS2), which combines the MD and PSD values of a given 24-2 VF test and categorizes it from normal – stage 0 through borderline to stages 1 to 5. Similar to the OCT metrics, we considered borderline (for GHT and GSS2) and less than 5% (for MDs and PSDs) results as abnormal.^34^ Note that the GSS2 is not readily available on HFA's VF reports, but it can be calculated relatively easily.

### Structure–Function Metrics

We also analyzed and estimated the performance of various combinations of structural–OCT and functional–VF metrics, that is, structure–function (S–F) agreement. We evaluated all possible combinations between G metrics from the cpRNFL and GCL and the MD and PSD of the 24-2 and 10-2. In addition, we assessed one of the criteria, described by Iyer et al., which combines sectoral cpRNFL and VF metrics.^15^ In particular, we estimated performance measures for their third criterion, which defines abnormality as “having matching abnormal OCT quadrant and GHT abnormalities,” here denoted as matching Q and GHT. Note that in their study, Iyer et al. used the superior or inferior quadrant, while Heidelberg's Spectralis divides each region into 2 sectors: temporal–superior and nasal–superior for superior, and TI and nasal–inferior for inferior. We looked for defect in either one of these sectors. For example, an abnormal temporal–superior or an abnormal nasal–superior sector was dealt as an abnormal superior region. Last, we evaluated combinations of our new S–S metric with the MD and the PSD of the 24-2 and 10-2 VFs.

### Performance Analysis

Specificity was estimated based on the number of HC eyes that each metric falsely identified as abnormal. These were considered clear FPs. Estimating sensitivity is more complicated owing to a lack of an accepted reference standard and the absence of a single test that can confirm the presence of glaucomatous damage. We defined 40 of the 61 suspect/glaucoma eyes as likely glaucoma (LG) based on our previously described method which evaluates the S–F agreement in an automated and objective fashion.^35^ In brief, probability and deviation values from the GCL+ and RNFL measures of a widefield OCT scan were overlaid by pattern deviation values from the 24-2 and 10-2 VF tests. When a VF location was abnormal at the 5% level and the corresponding structural region was abnormal at the 10% level, then this location was considered as showing abnormal structure–abnormal function (aS–aF) agreement. An eye is considered abnormal if the number of locations with aS–aF agreement exceeds a given threshold. We have previously reported on the high diagnostic performance of this method as well as its superiority against other commonly used summary metrics.^33^ To increase the likelihood that a LG eye is a true positive, we added 2 additional criteria. First, we set the minimum number of aS–aF locations required for an eye to be considered abnormal to 3 aS–aF locations, instead of the 2 locations as in the original study.^35^ Second, we required a replication; that is, 2 consecutive baseline OCT-VF tests had to show aS–aF agreement, with at least 3 aS–aF locations in each OCT–VF pair. Forty patient eyes satisfied these criteria. The average (± standard deviation) number of aS–aF locations per eye was 19 ± 16 (range, 3–64 locations). The sensitivity of all the metrics was determined by their identification of these 40 LG eyes as abnormal.

Similar data for these OCT and VF metrics, and their combinations, were collected from the second baseline date to assess the repeatability of performance measures. In addition, OCT and VF metrics from both baseline dates were combined to estimate 95% confidence intervals for specificity, sensitivity, and accuracy using bootstrapping (resampling with replacement and 1000 iterations). Last, the first and second baseline OCT and VF data were collected from the same study eyes using a different commercial OCT instrument (Topcon Inc., Tokyo, Japan) to assess the reproducibility of our findings with a different OCT instrument.

## Results

### OCT and S–S Metrics

In general, OCT metrics, when used in isolation, miscategorized between 1 and 6 HC eyes as abnormal (FP) and 7 to 32 of the 40 LG eyes as normal (false negative). For example, the most commonly used G_small_ metric, as shown in red in Table 1, had 5 FPs, for a 91% specificity, although it detected only 28 of the 40 LG eyes (70%). The best performing single OCT metrics were the TI region of the cpRNFL (TI_small_) and the GCL (TI_GCL_), as shown in bold and dashed underline in Table 1. They both had an acceptable specificity of approximately 95% and the highest specificities (91% and 87%). However, they still failed to detect a relatively large number of these LG eyes, with the TI_small_ sector missing 7 (17%) and the TI_GCL_ missing 10 (25%) LG eyes. The G_large_ and sectoral metrics from the large (4.7 mm) circle scan showed similar levels of specificity, but sensitivity was markedly decreased compared with the cpRNFL_small_, BMO–MRW, and GCL metrics. The same was true for other metrics deriving from the posterior pole cube scan, and more specifically the IPL (i.e., G_IPL_ and sectors) and those calculated from total retinal thickness (i.e., G_Retina_ and sectors). For reference, we provide those results in Supplementary Table S6.

**Table 1.** Performance Measures of Single OCT Metrics

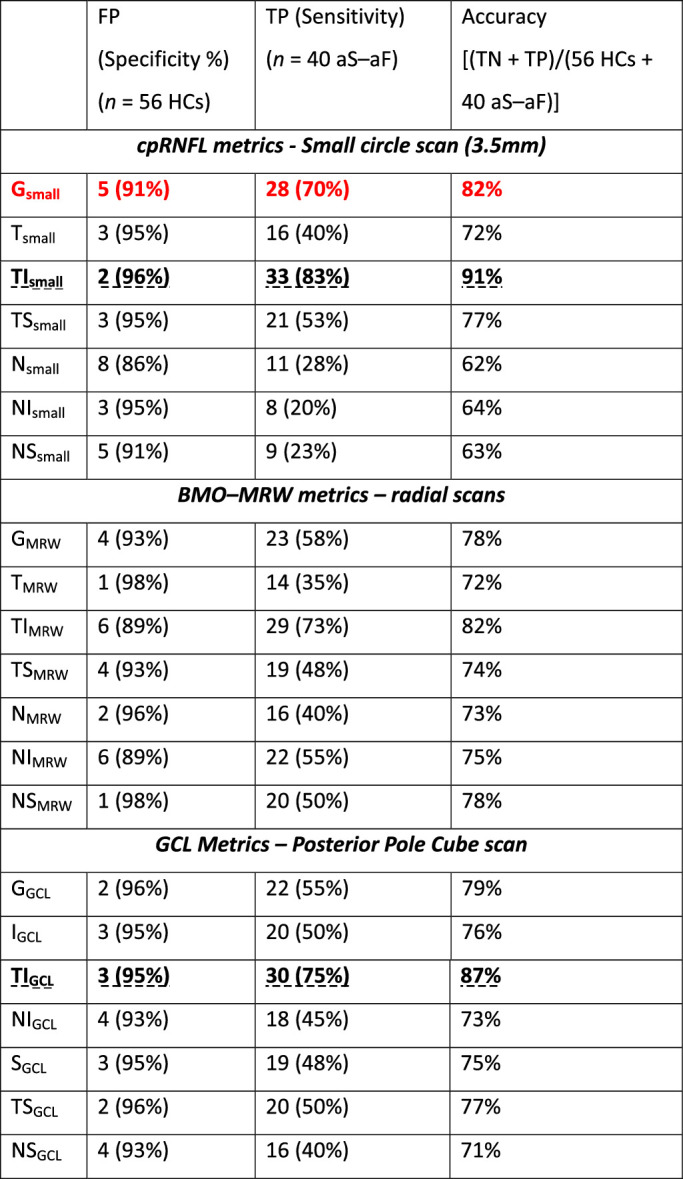

FP, false positive; TN, true negative; TP, true positive.

The combination of cpRNFL_small_ metrics with either BMO-MRW or GCL markedly decreased the number of FPs, improving specificity (Table 2). For example, the pairing of the G metrics from the cpRNFL and the BMO–MRW measures ({G_small_ AND G_MRW_}, highlighted with green in Table 2) misclassified only 2 HC eyes (i.e., 96% specificity). Even better, the combination of the TI region from the same measures ({TI_small_ AND TI_MRW_}, noted with bold and dashed underline in Table 2) falsely detected as abnormal only 1 HC eye. However, these combinations markedly reduced sensitivity with only 15 of 40 (38%) and 26 of 40 (65%) LG eyes detected by the G and TI combinations, respectively. The criterion from Yang et al. ({Any (1_small_ + 1_MRW_)}, dark red and double underline in Table 2), which effectively looks for the same cpRNFL/BMO–MRW agreement in any of the 6 paired sectors instead of just the TI region, correctly identified 3 more LG eyes, for a total of 29 TPs.^14^ However, this improvement in sensitivity (from 65% to 73%) came at the cost of decreased specificity, to 93% with 4 FPs from the HC group.

**Table 2.** Performance Measures of Combinations of OCT Metrics

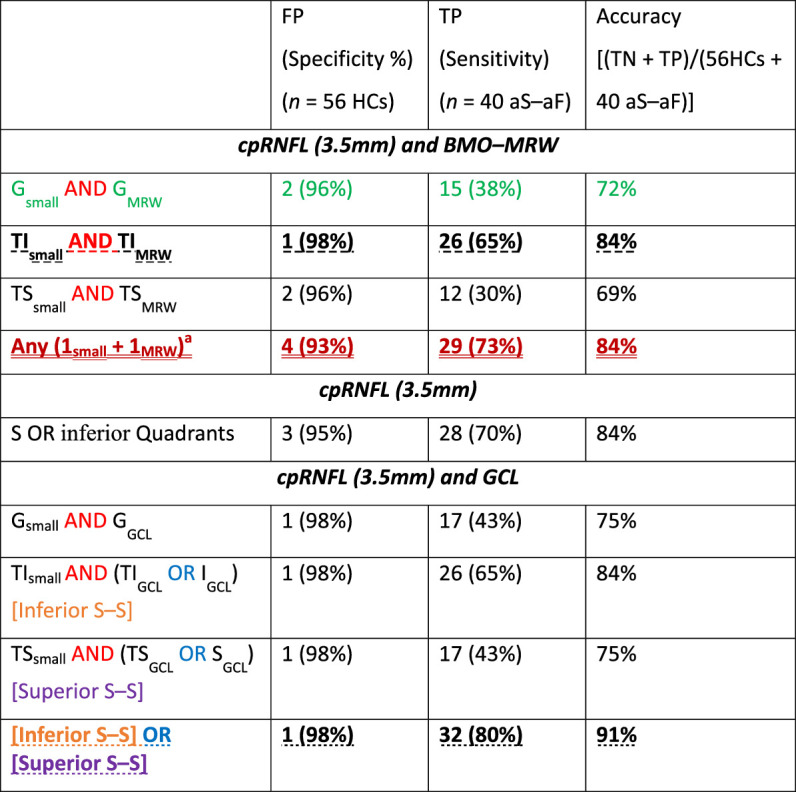

FP, false positive; TN, true negative; TP, true positive.

^a^Yang et al.^14^

The best performing combination of OCT metrics was our new S–S metric, ({[Inferior S–S] OR [Superior S–S]}, bottom row in Table 2**)**. It falsely categorized only 1 HC eye as abnormal, and it detected 32 of the 40 LG eyes (i.e., 80% sensitivity). In general, it had one of the highest accuracies, at 91%, among the single or combined OCT metrics.

### VF and F–F Metrics

It is well-documented that the variability of summary metrics in VF testing is high.^27^^,^^36^^–^^38^ As shown in Table 3, single and combined VF metrics performed poorly, mostly by causing an excessive number of FPs from the HC group.

**Table 3.** Performance Measures of 24-2 and 10-2 VF Metrics, in Isolation and in Combinations (i.e., F–F agreement)

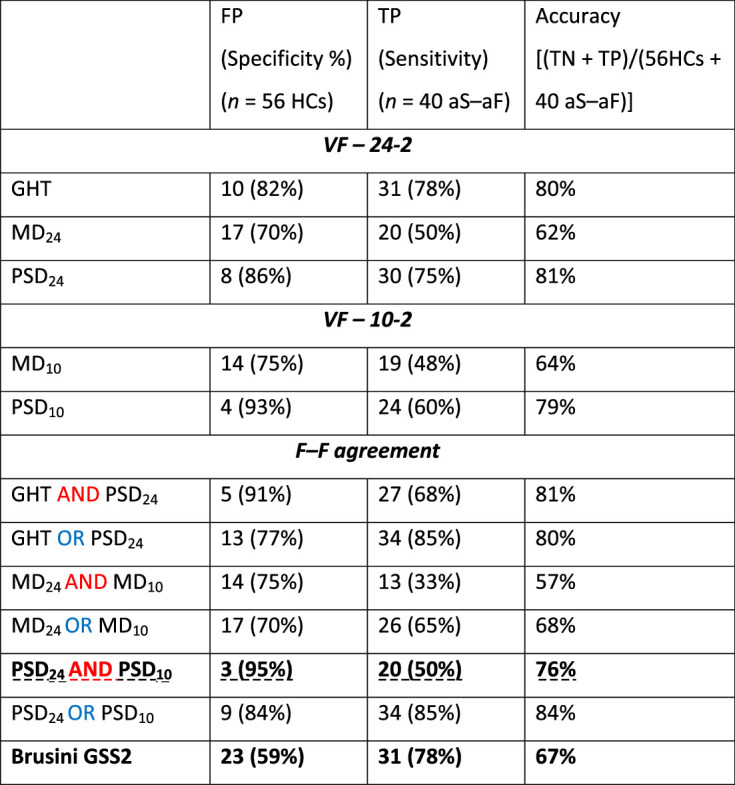

FP, false positive; TN, true negative; TP, true positive.

The combination of VF metrics, such as for example the PSD measures from the 24-2 and the 10-2 ({PSD_24-2_ AND PSD_10-2_}, bold and dashed underline in Table 3), slightly improved specificity to more acceptable levels (95%, 3 FPs), although it detected only half of the 40 LG eyes. Finally, the GSS2 (in bold, last row in Table 3), which combines MD and PSD values, from the 24-2 test, in a nonlinear fashion correctly identified 31 of the 40 LG eyes, although it had the poorest specificity (59%) with 23 FPs.^34^

### S–F Metrics

The combination of structural (OCT) and functional (VF) metrics presented the highest specificity (Table 4). For example, asking whether the {G_small_ OR the G_GCL_} metrics are abnormal and seeking confirmation from the {PSD_24-2_ OR PSD_10-2_} metric (see bold and dashed undereline in Table 4), yielded only 1 FP (i.e., 98% specificity). Our new S–S metric combined with the {PSD_24-2_ OR PSD_10-2_} metric (last row in Table 4) had a specificity of 100%. A similarly high specificity was achieved by looking for matching abnormalities in the {Q and GHT} metric (third criterion by Iyer et al.; green and double underline in Table 4).^15^ Yet, all of them failed to detect many of the LG eyes, with only 28 (70%), 26 (65%), and 19 (48%) TPs, respectively.

**Table 4.** Performance Measures of Combinations Between OCT-VF Summary Metrics (i.e., S–F agreement)

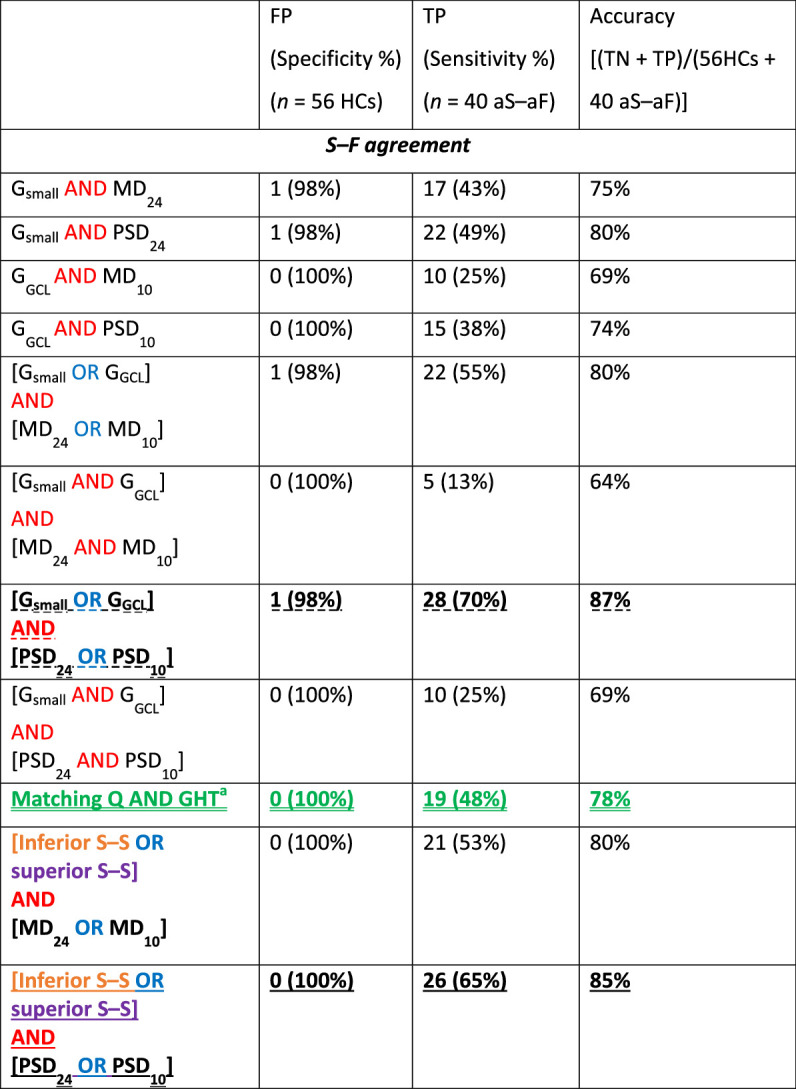

FP, false positive; TN, true negative; TP, true positive.

^a^Third criterion from Iyer et al.^15^

### Repeatability and Reproducibility

Performance measures for all OCT and VF summary metrics based on the second baseline test were almost identical to those reported from the first test. Table 5 shows the specificity, sensitivity and accuracy of the best performing metric in Tables 1, 2, and 4 for first and second baseline tests.

**Table 5.** Performance Measures From 2 different Baseline Test Dates for OCT Spectralis’ Top 3 Best Performing metrics and Performance Measures From Same Dates for OCT Topcon's Metrics That Are Similar

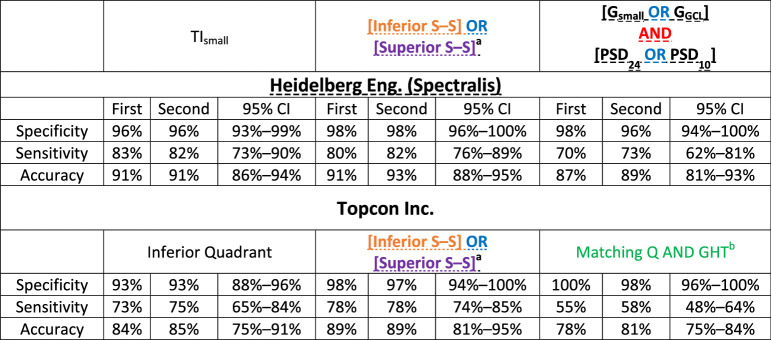

CI, confidence interval.

^a^[Inferior S–S] OR [Superior S–S] for OCT Topcon is based on the Inferior (S) and Superior (S) quadrant, instead of the TI and temporal–superior sectors for OCT Heidelberg.

^b^Third criterion from Iyer et al.^15^

The analysis of OCT summary metrics based on a Topcon Inc. instrument, including their pairing with VF metrics showed similar performance measures and led to similar conclusions. Table 5 (bottom half) shows performance measures and their 95% CIs for the metrics that are equivalent, but not identical, to the HE metrics presented on the upper half of the table.

## Discussion

Glaucoma specialists routinely examine whether metrics (summary statistics), provided in OCT and VF commercial reports, are within or outside normal limits to inform their decisions regarding the presence of glaucomatous damage. However, we, among others, have argued that these summary metrics can misguide clinicians and falsely categorize HCs and glaucomatous eyes more often than it is generally believed.^21^^,^^22^^,^^30^^,^^39^^,^^40^ However, other recent studies have proposed new combinations of these metrics in an attempt to improve their performance.^14^^,^^15^^,^^34^ Because different studies use different metrics, inclusion criteria, and reference standards, it is difficult to reconcile the results of these studies. In the present study, we evaluated the detection performance of a wide variety of metrics, based on summary statistics reported in commercially available OCT and VF reports. In addition, we investigated the effect on these performance measures when they are grouped, either based on the same modality (i.e., S–S or function–function), as well as S–F pairings. Given that any summary metric, more or less, can succeed in identifying severe, and even moderate, glaucoma, we studied eyes classified as early glaucoma, based on a 24-2 MD better than −6 dB. To avoid including eyes with an uncertain diagnosis, we restricted our measure of sensitivity to 40 eyes classified as LG, based on an automated and objective method and our measure of specificity with 56 HC eyes recruited with a normal intraocular pressure and fundus examination.^33^^,^^35^

### All Metrics Make Mistakes

All OCT and VF summary metrics, in isolation and in combinations, failed to detect all 40 LG eyes. None of the metrics with a specificity of better than 95% had sensitivities of better than 83%. The most commonly used OCT and VF summary metrics, the G_small_, GHT or PSD_24_, not only missed 25% to 30% of the LG eyes with glaucomatous damage, but they also falsely classified a relatively high number of HCs as abnormal (see Tables 1 and 3). Even the newly proposed summary metrics combining BMO–MRW and cpRNFL measures or OCT and 24-2 VF, had sensitivities of less than 80%.^14^^,^^15^ Our proposed S–S metric was 1 of 2 metrics with the highest accuracy, 91%, although it too had a modest sensitivity of 80%.

There are at least 3 factors that limit the accuracy of OCT summary metrics. First, segmentation algorithms make mistakes and correcting them is difficult in general, and typically not feasible in a clinical practice. In fact, we and others have shown that subtle segmentation errors, which are difficult to detect, are common, and can lead to false diagnoses from the OCT summary metrics.^41^^–^^43^ Second, if the scan is not placed properly on the disc or foveal center it can too lead to false diagnoses.^44^ Third, early glaucomatous damage often involves relatively local defects that are missed by summary statistics, which include regions larger than these defects.^45^ A post hoc analysis of the mistakes based on each summary metric showed that segmentation errors and local damage were the most common reasons for FPs and false negatives.

### The Importance of the TI Region and Macular Damage

Interestingly, the other metric with the highest (91%) accuracy was TI_small_, the inferior temporal region of the small circle scan. Note that TI represents only about 45° of the circle scan and is associated with less than one-half of the macular region. This is the circumpapillary region that corresponds to what we have called the macular vulnerability zone.^20^ The relatively good performance of this single metric, TI, further highlights how often glaucomatous damage involves the macula.^19^

### The Advantage of Topographic Agreement

We have previously discussed the benefits of seeking topographic agreement either between 2 OCT measures (i.e., RNFL and macular GCL) or between structural (OCT) and functional (VF) measures.^32^^,^^33^ The results of the present study are in agreement. For example, our new S–S metric and the {Matching Q and GHT}, an S–F metric by Iyer et al., correctly classified all HC eyes (100% specificity).^15^ As expected, however, this improvement in specificity came at some cost to sensitivity, with the S–S metric and the {Matching Q and GHT} missing 20% and more than 50% of the LG eyes, respectively.

There was little overlap between the S–S metric and the {Matching Q and GHT} results. Fourteen LG eyes were not detected by the {Matching Q and GHT} metric, but were correctly classified as glaucomatous by the S–S metric, although 1 LG eye was not detected by the S–S metric, but was correctly classified by the {Matching Q and GHT} metric.

### Clinical Relevance

Detection methods that are highly specific are important for clinical and research purposes, because they have a lesser likelihood in sending healthy eyes to the glaucoma clinic or recruiting participants in clinical trials who do not actually have glaucomatous damage. Most combinations of OCT and VF metrics achieved high specificity; they misclassified either none or 1 HC eye (see Tables 2 and 4). Although the best sensitivity was only 80% for our new S–S metric and 83% for TI_small_, the eyes missed had relatively early glaucoma. Thus, these metrics should do considerably better when screening for moderate and advanced glaucoma. In fact, because these metrics are based solely on OCT, they may prove to be useful for screening large numbers of patients for moderate or advanced glaucomatous damage.

It is evident, however, from the results of this study that some eyes with early glaucoma will be missed. These misses tend to be eyes with defects near fixation or localized damage (shown on the OCT and/or VF) and they often show normal (i.e., green) summary metrics of RNFL or GCL thickness.^19^ Therefore, any glaucoma detection method that solely relies on the use of OCT and/or VF summary metrics will miss these clinically important defects.

### Limitations

The fact that we did not manually correct the segmentation of OCT circle and radial scans might have affected the detection performance of OCT metrics, which could be considered a limitation of this study. This point is particularly important when considering the results of this study in comparison to those presented by Yang et al.^14^ However, we opted not to apply any changes to the automated measures so as to be more representative of the real-life clinical practice.

Second, the results here should be replicated with a larger sample and a different site, although the repeat reliability and the replication with an OCT instrument from another manufacturer suggests the findings are robust.

Third, the results of studies such as this one will be highly dependent on the Reference Standard (RS) chosen, and there is no generally accepted structure–function definition of glaucoma. Our RS definition of LG relied on an automated and objective method that evaluated S–F agreement. We chose this RS to ensure that all LG eyes were highly likely to have glaucomatous damage. In other words, we believe that these 40 LG eyes, and the 56 HCs, should not be missed by any metric. It is, however, worth noting that the reported sensitivity and specificity are highly dependent on our choice of RS, and a less stringent criteria would improve sensitivity, at the cost of decreased specificity.

Last, our primary study purpose was the evaluation of OCT and VF metrics that can be accessible to clinicians via commercially available OCT and VF reports. In addition, we included combinations of metrics that can be easily applied or calculated from these metrics. As a result, we did not include possible metrics that are more complicated in their estimation or computationally intensive. It is unlikely that any metrics, new or old, will deviate significantly from the general findings of this study. For example, one VF metric that was not reported here is a PSD measure of the 12 most-central points of the 24-2 (C24-2), proposed by Wu et al.^46^ In fact, we have previously shown that the C24-2 metric, like the PSD_10_ metric, will miss eyes with central glaucomatous damage, clearly identified in the total and pattern deviation maps.^47^ Another example, and for an OCT metric, previous studies have shown that the ganglion cell complex has better repeatability as compared with other macular summary metrics and greater discriminability for both the detection and progression of glaucomatous damage, especially in cases with moderate to advanced glaucoma.^48^^–^^51^ Ganglion cell complex summary metrics were not calculated in this study because they were not readily available. It is likely, however, that they would show similar detection performance with the GCL and GCL+ metrics.

## Conclusions

A detection method that relies solely on the use of single OCT or VF metrics leads to the misidentification of some HC eyes as glaucomatous and fails to detect some eyes with early glaucomatous damage. Combinations of OCT and VF metrics that look for S–S and S–F agreement can decrease the number of FPs and be highly specific, which has important implications for screening purposes. However, the most accurate combination was a new S–S metric, that seeks agreement between cpRNFL and GCL loss at a hemifield level, with a specificity of 98%, but a sensitivity of only 80% for eyes with early glaucoma. Last, the detection issues from all metrics presented in this study argue for a topographic comparison of abnormal regions on VF and OCT and careful inspection of actual OCT scan images.

## Supplementary Material

Supplement 1
