# Supplementary material for: Detection of Early Glaucomatous Damage: Performance of Summary Statistics From Optical Coherence Tomography and Perimetry
Source: Transl Vis Sci Technol. 2022 Mar 30;11(3):36. doi: 10.1167/tvst.11.3.36 (PMC8976935; doi:10.1167/tvst.11.3.36)
Supplement: Supplement 1 [file tvst-11-3-36_s001.pdf]

Table S1: A list of the summary metrics and other important abbreviations

**Naming Convention: X_Z**	**X denotes the summary metric**	**Z denotes the source**
G_{small}	Global	3.5mm circle scan
T_{small}	Temporal	3.5mm circle scan
TI_{small}	Temporal Inferior	3.5mm circle scan
TS_{small}	Temporal Superior	3.5mm circle scan
N_{small}	Nasal	3.5mm circle scan
NI_{small}	Nasal Inferior	3.5mm circle scan
NS_{small}	Nasal Superior	3.5mm circle scan
G_{MRW}	Global	Radial Scans – Minimum Rim Width
T_{MRW}	Temporal	Radial Scans – Minimum Rim Width
TI_{MRW}	Temporal Inferior	Radial Scans – Minimum Rim Width
TS_{MRW}	Temporal Superior	Radial Scans – Minimum Rim Width
N_{MRW}	Nasal	Radial Scans – Minimum Rim Width
NI_{MRW}	Nasal Inferior	Radial Scans – Minimum Rim Width
NS_{MRW}	Nasal Superior	Radial Scans – Minimum Rim Width
G_{GCL}	Global	Posterior Pole Scan – Ganglion Cell Layer
I_{GCL}	Inferior	Posterior Pole Scan – Ganglion Cell Layer
TI_{GCL}	Temporal Inferior	Posterior Pole Scan – Ganglion Cell Layer
NI_{GCL}	Nasal Inferior	Posterior Pole Scan – Ganglion Cell Layer
S_{GCL}	Superior	Posterior Pole Scan – Ganglion Cell Layer
TS_{GCL}	Temporal Superior	Posterior Pole Scan – Ganglion Cell Layer
NS_{GCL}	Nasal Superior	Posterior Pole Scan – Ganglion Cell Layer
GHT	Glaucoma Hemifield Test	24-2 Visual Field
MD_{24}	Mean Deviation	24-2 Visual Field
PSD_{24}	Pattern Standard Deviation	24-2 Visual Field
MD_{10}	Mean Deviation	10-2 Visual Field
PSD_{10}	Pattern Standard Deviation	10-2 Visual Field
Q and GHT	Quadrant and Glaucoma Hemifield Test	Q: 3.5mm circle scan, GHT: 24-2 Visual Field
	**Other Important Abbreviations**	
**Abbreviation**	**Explanation**	**Comment**
RNFL	Retinal Nerve Fiber Layer	
cpRNFL	Circumpapillary RNFL	
GCL	Ganglion Cell Layer	Available on Heidelberg Probability Maps
GCL+	Ganglion Cell + Inner Plexiform Layer	Available on Topcon Probability Maps
MRW	Minimum Rim Width	
cpRNFL	Circumpapillary RNFL	
S-S	Structure-Structure	It implies S-S correspondence/agreement
S-F	Structure-Function	It implies S-F correspondence/agreement

Table S2: Performance Measures of Single OCT Metrics for a stricter criterion (Abnormal = 'Outside Normal Limits'); FP: False Positive, TP: True Positive, TN: True Negative, HC: Healthy Control, aS-aF: abnormal Structure – abnormal Function

	FP (Specificity %) [n = 56 HCs]	TP (Sensitivity) [n = 40 aS-aF]	Accuracy [(TN + TP) / (56HCs + 40 aS-aF)]
cRNFL metrics - Small circle scan (3.5mm)			
G_{small}	1 (98%)	17 (43%)	75%
T_{small}	1 (98%)	7 (18%)	65%
TI_{small}	2 (96%)	22 (55%)	79%
TS_{small}	1 (98%)	10 (25%)	68%
N_{small}	4 (93%)	5 (13%)	59%
NI_{small}	1 (98%)	3 (8%)	60%
NS_{small}	0 (100%)	4 (10%)	63%
BMO-MRW metrics – Radial scans			
G_{MRW}	0 (100%)	10 (25%)	69%
T_{MRW}	1 (98%)	7 (18%)	65%
TI_{MRW}	1 (98%)	21 (53%)	79%
TS_{MRW}	1 (98%)	7 (18%)	65%
N_{MRW}	0 (100%)	8 (20%)	67%
NI_{MRW}	1 (98%)	14 (35%)	72%
NS_{MRW}	0 (100%)	4 (10%)	63%
GCL Metrics – Posterior Pole Cube scan			
G_{GCL}	0 (100%)	14 (35%)	73%
I_{GCL}	0 (100%)	15 (38%)	74%
TI_{GCL}	1 (98%)	24 (60%)	82%
NI_{GCL}	0 (100%)	13 (33%)	72%

S_{GCL}	2 (96%)	14 (35%)	71%
TS_{GCL}	0 (100%)	14 (35%)	73%
NS_{GCL}	0 (100%)	9 (23%)	68%

Table S3: Performance measures of combinations of OCT metrics for a stricter criterion (Abnormal = 'Outside Normal Limits'). FP: False Positive, TP: True Positive, TN: True Negative, HC: Healthy Control, aS-aF: abnormal Structure – abnormal Function

	FP (Specificity %) [n = 56 HCs]	TP (Sensitivity) [n = 40 aS-aF]	Accuracy [(TN + TP) / (56HCs + 40 aS-aF)]
cRNFL (3.5mm) and BMO-MRW			
G_{small} AND G_{MRW}	0 (100%)	9 (23%)	68%
TI_{small} AND TI_{MRW}	0 (100%)	16 (40%)	75%
TS_{small} AND TS_{MRW}	0 (100%)	4 (10%)	63%
cRNFL (3.5mm) and GCL			
G AND G_{GCL}	0 (100%)	9 (23%)	68%
TI AND (TI_{GCL} OR I_{GCL}) [Inferior S-S]	0 (100%)	17 (43%)	76%
TS AND (TS_{GCL} OR S_{GCL}) [Superior S-S]	1 (98%)	8 (20%)	66%
**[Inferior S-S] OR [Superior S-S]**	1 (98%)	**19 (48%)**	**77%**

Table S4: Performance measures of 24-2 and 10-2 VF metrics, in isolation and in combinations (i.e., function-function, F-F, agreement), for a stricter criterion (Abnormal = 'Outside Normal Limits' or P <2%). FP: False Positive, TP: True Positive, TN: True Negative, HC: Healthy Control, aS-aF: abnormal Structure – abnormal Function

	FP (Specificity %) [n = 56 HCs]	TP (Sensitivity) [n = 40 aS-aF]	Accuracy [(TN + TP) / (56HCs + 40 aS-aF)]
Visual Field – 24-2			
GHT	4 (93%)	25 (63%)	80%
MD_{24-2}	5 (91%)	11 (28%)	65%
PSD_{24-2}	1 (98%)	18 (45%)	76%
Visual Field – 10-2			
MD_{10-2}	3 (95%)	8 (20%)	64%
PSD_{10-2}	2 (96%)	12 (30%)	69%
F-F Combination			
GHT AND PSD_{24-2}	5 (91%)	18 (45%)	72%
GHT OR PSD_{24-2}	13 (77%)	25 (63%)	71%
MD_{24-2} AND MD_{10-2}	2 (96%)	2 (5%)	58%
MD_{24-2} OR MD_{10-2}	6 (89%)	17 (43%)	70%
PSD_{24-2} AND PSD_{10-2}	1 (98%)	8 (20%)	66%
PSD_{24-2} OR PSD_{10-2}	2 (96%)	22 (55%)	79%
Brusini GSS2	11 (80%)	22 (55%)	70%

Table S5: Performance measures of combinations between OCT-VF summary metrics (i.e., structure-function, S-F, agreement), for a stricter criterion (Abnormal = 'Outside Normal Limits'). FP: False Positive, TP: True Positive, TN: True Negative, HC: Healthy Control, aS-aF: abnormal Structure – abnormal Function

	FP (Specificity %) [n = 56 HCs]	TP (Sensitivity) [n = 40 aS-aF]	Accuracy [(TN + TP) / (56HCs + 40 aS-aF)]
S-F Combination			
G_{small} AND MD 24-2	0 (100%)	6 (15%)	65%
G_{small} AND PSD 24-2	0 (100%)	9 (23%)	68%
G_{GCL} AND MD 10-2	0 (100%)	5 (13%)	64%
G_{GCL} AND PSD 10-2	0 (100%)	7 (18%)	66%
**[G_{small} OR G_{GCL}] AND [MD_{24} OR MD_{10}]**	0 (100%)	12 (30%)	71%
[G_{small} AND G_{GCL}] AND [MD_{24} AND MD_{10}]	0 (100%)	0 (0%)	58%
**[G_{small} OR G_{GCL}] AND [PSD_{24} OR PSD_{10}]**	0 (100%)	15 (38%)	74%
[G_{small} AND G_{GCL}] AND [PSD_{24} AND PSD_{10}]	0 (100%)	2 (5%)	60%
**[Inferior S-S] OR [Superior S-S] AND [MD_{24} OR MD_{10}]**	0 (100%)	11 (28%)	70%
**[Inferior S-S] OR**	0 (100%)	15 (38%)	74%

[Superior S-S]<br>AND<br>[PSD_{24} OR PSD_{10}]			

Table S6: Performance measures from summary metrics that are not commonly used, i.e., the circumpapillary retinal nerve fiber layer thickness from the large (4.7mm) circle scan, and the inner plexiform layer (IPL) and the total retinal thickness from the posterior pole scan. FP: False Positive, TP: True Positive, TN: True Negative, HC: Healthy Control, aS-aF: abnormal Structure – abnormal Function

	Abnormal = Borderline or ONL			Abnormal = ONL		
	FP (Specificity %) [n = 56 HCs]	TP (Sensitivity) [n = 40 aS-aF]	Accuracy [(TN + TP) / (56HCs + 40 aS-aF)]	FP (Specificity %) [n = 56 HCs]	TP (Sensitivity) [n = 40 aS-aF]	Accuracy [(TN + TP) / (56HCs + 40 aS-aF)]
cRNFL metrics - Large circle scan (4.7mm)						
G_{large}	3 (94%)	15 (46%)	74%	1 (98%)	11 (33%)	71%
T_{large}	1 (98%)	10 (30%)	70%	1 (98%)	6 (18%)	65%
TI_{large}	4 (92%)	26 (79%)	86%	1 (98%)	17 (52%)	79%
TS_{large}	2 (96%)	18 (55%)	79%	1 (98%)	6 (18%)	65%
N_{large}	5 (89%)	7 (21%)	61%	2 (96%)	3 (9%)	60%
NI_{large}	2 (96%)	4 (12%)	61%	0 (100%)	3 (9%)	63%
NS_{large}	3 (94%)	3 (9%)	59%	0 (100%)	1 (3%)	60%
IPL Metrics – Posterior Pole Cube scan						
G_{IPL}	2 (96%)	16 (40%)	73%	0 (100%)	13 (33%)	72%
I_{IPL}	3 (95%)	19 (48%)	75%	2 (96%)	13 (33%)	70%
TI_{IPL}	2 (96%)	22 (55%)	79%	0 (100%)	20 (50%)	79%
NI_{IPL}	3 (95%)	16 (40%)	72%	0 (100%)	10 (25%)	69%
S_{IPL}	3 (95%)	15 (38%)	71%	1 (98%)	9 (23%)	67%
TS_{IPL}	2 (96%)	15 (38%)	72%	0 (100%)	10 (25%)	69%
NS_{IPL}	1 (98%)	14 (35%)	72%	0 (100%)	8 (20%)	67%
Total Retina Metrics – Posterior Pole Cube scan						
G_{RET}	3 (95%)	12 (30%)	68%	0 (100%)	6 (15%)	65%

I_{RET}	3 (95%)	13 (33%)	69%	0 (100%)	6 (15%)	65%
TI_{RET}	2 (96%)	15 (38%)	72%	0 (100%)	8 (20%)	67%
NI_{RET}	3 (95%)	11 (28%)	67%	0 (100%)	6 (15%)	65%
S_{RET}	5 (91%)	10 (25%)	64%	1 (98%)	4 (10%)	62%
TS_{RET}	1 (98%)	9 (23%)	67%	0 (100%)	5 (13%)	64%
NS_{RET}	4 (93%)	10 (25%)	65%	0 (100%)	3 (8%)	62%
